# Generational perspective on asthma self‐management in the Bangladeshi and Pakistani community in the United Kingdom: A qualitative study

**DOI:** 10.1111/hex.13579

**Published:** 2022-08-23

**Authors:** Salina Ahmed, Hilary Pinnock, Anna Dowrick, Liz Steed

**Affiliations:** ^1^ Centre for Primary Care, Wolfson Institute of Population Health Queen Mary University of London London UK; ^2^ School of Health Sciences University of Greenwich London UK; ^3^ Usher Institute, College of Medicine and Veterinary Science The University of Edinburgh Edinburgh UK; ^4^ Nuffield Department of Primary Care Health Sciences, Medical Sciences Division University of Oxford Oxford UK

**Keywords:** acculturation, asthma, culture, generation, qualitative, self‐management, South Asians

## Abstract

**Background:**

Self‐management strategies improve asthma outcomes, although interventions for South Asian populations have been less effective than in White populations. Both self‐management and culture are dynamic, and factors such as acculturation and generation have not always been adequately reflected in existing cultural interventions. We aimed to explore the perspectives of Bangladeshi and Pakistani people in the United Kingdom, across multiple generations (first, second and third/fourth), on how they self‐manage their asthma, with a view to suggesting recommendations for cultural interventions.

**Methods:**

We purposively recruited Bangladeshi and Pakistani participants, with an active diagnosis of asthma from healthcare settings. Semi‐structured interviews in the participants' choice of language (English, Sylheti, Standard Bengali or Urdu) were conducted, and data were analysed thematically.

**Results:**

Twenty‐seven participants (13 Bangladeshi and 14 Pakistani) were interviewed. There were generational differences in self‐management, influenced by complex cultural processes experienced by South Asians as part of being an ethnic minority group. Individuals from the first generation used self‐management strategies congruent to traditional beliefs such as ‘sweating’ and often chose to travel to South Asian countries. Generations born and raised in the United Kingdom learnt and experimented with self‐management based on their fused identities and modified their approach depending on whether they were in familial or peer settings. Acculturative stress, which was typically higher in first generations who had migration‐related stressors, influenced the priority given to asthma self‐management throughout generations. The amount and type of available asthma information as well as social discussions within the community and with healthcare professionals also shaped asthma self‐management.

**Conclusions:**

Recognizing cultural diversity and its influence of asthma self‐management can help develop effective interventions tailored to the lives of South Asian people.

**Patient or Public Contribution:**

Patient and Public Involvement colleagues were consulted throughout to ensure that the study and its materials were fit for purpose.

## 1 INTRODUCTION

Asthma is a variable respiratory illness, heterogeneous in its presentation, aetiology and underlying inflammation.^1^ The Lancet Commission and clinical guidelines emphasize that self‐management is a core aspect of managing asthma that helps individuals deal with its variable nature. It includes undertaking asthma education, using written personalized asthma action plans and participating in clinical discussions and support.^1^, ^2^, ^3^ Self‐management can be defined as ‘The tasks that individuals must undertake to live with one or more chronic conditions. These tasks include having the confidence to deal with medical management, role management, and emotional management of their conditions’.^4^ This definition reflects a shift away from the idea of the healthcare professional (HCP) as the sole expert, to a more collaborative approach, where both HCPs and patients share responsibility and expertise in achieving successful self‐management and improved asthma outcomes,^5^, ^6^ although the meaning attributed to self‐management is not universal, but individually and socially constructed, varying according to the given context and generation.^6^, ^7^, ^8^ People manage their own condition(s) all the time, fluctuating from active to passive participation, thereby bringing distinct experiences and expertise to their self‐management.^9^, ^10^ South Asians in the United Kingdom benefit less from most existing asthma self‐management interventions, compared to White populations.^11^, ^12^ Despite increasing awareness that neither culture nor self‐management are static, intervention efforts to engage with different cultural groups are often limited to simplistic strategies such as using culturally relevant images or one‐off translation of written materials.^12^, ^13^, ^14^ This advocates for a more nuanced understanding of self‐management among South Asians from ethnic minority communities across generations.

Contextualized understanding of self‐management is likely to be important for South Asians from ethnic minority groups, which can then be translated to improved interventions that consider how cultural characteristics, such as acculturation, and self‐management are translated and actioned within one's sociocultural context.^15^, ^16^, ^17^ Acculturation is the process where cultural changes are influenced by encountering another mainstream culture in a new environment over time and generation. Generally, the first migrant generation adapt to the ‘separation’ style of acculturation, where they maintain their own culture and limit adaptations to the mainstream culture, due to difficulties with language, tradition, lifestyle, historical and other influences. South Asians born in Western countries (second generation or beyond) are more inclined to adapt to the ‘integration’ strategy, where they maintain their own original culture and integrate with the mainstream culture, such as speaking in English, having a broad peer network and a fused sense of ethnic and national identity (see Box 1).^20^, ^21^, ^22^, ^23^, ^24^, ^25^ In addition, acculturation encompasses acculturative stress, which is the daily reactions, experiences and psychological and sociocultural adaptation challenges encountered in a new or different mainstream society. Higher acculturative stress, for instance, might be experienced in first generations because of the loss of social, cultural and economic capital from migration, such as the absence of social support.^26^, ^27^

Box 1. **Acculturation strategies adapted from Berry's acculturation model^18^, ^19^**

The acculturation model suggests that individuals or groups can freely adapt to a new or mainstream environment in one of four ways, over time or generations, and its flexible nature can be dependent on a given situation. These four adaptations are known as acculturation strategies:

1. Integration: Individuals who maintain their original culture and also integrate with the mainstream culture
2. Assimilation: Individuals who disconnect from their original culture to fit in with the mainstream culture
3. Separation: Individuals who hold on to their original culture and avoid any adaptation or contact with the mainstream culture
4. Marginalization: Individuals who lose maintenance and contact with both original and mainstream cultures

The reaction and experience of daily stress and the degree of psychological and/or sociocultural adaptation challenges resulting from encountering a larger mainstream society are known as acculturative stress.

These acculturative contextual influences suggest that there are intergenerational variations in cultivating strategies for self‐managing asthma by reacting to the environment around them.^18^ Illness representations of asthma, for instance, can be guided by cultural influences that alter in accordance with experiences of education and migration.^10^, ^28^ However, the needs and preferences of self‐management across generations are rarely recognized within healthcare services, frequently relying on language adjustments in interventions and policies, which is not a culturally competent approach.^12^, ^29^ There has also been a tendency to focus primarily on the disease management side of self‐management, such as medicine adherence, and overlook psychosocial and cultural factors important for people to improve their asthma.^5^, ^12^, ^30^ This study aimed to explore the generational perspective of individuals from two South Asian subcultures—the Bangladeshi and Pakistani communities in the United Kingdom—on how they perceive and self‐manage their asthma, and how culture influences this.

## 2 MATERIALS AND METHODS

This study gained NHS ethical approval from South Yorkshire Research Ethics Committee (IRAS ID: 200955), governance approval from the Health Research Authority (23 January 2017; REC: 16/YH/0524) and sponsorship by Barts Health NHS Trust. All participants provided written/oral informed consent.

### 2.1 Study design and setting

The study involved one‐to‐one semi‐structured interviews of participants living in three ethnically diverse boroughs of London in the United Kingdom: Tower Hamlets, Newham and Waltham Forest. Participants were recruited from both primary care (three GP practices) and secondary care (three hospital asthma clinics, including one tertiary service for individuals with severe asthma). GP services provide most of the asthma care for patients registered with their practice, including diagnosis, routine reviews, supporting self‐management and acute care. Secondary care asthma clinics support the management of patients with diagnostic or treatment queries, and the acute and follow‐up care of severe asthma attacks, such as those requiring admission. The tertiary care severe asthma clinics specialize in the holistic and complex care of patients with ‘difficult to control’ asthma including those at risk of life‐threatening attacks. In addition, the study was advertised through a recruitment poster that was placed in healthcare settings, social media and community organizations such as sixth form schools and colleges, local community centres and grocery stores, as well as Queen Mary, University of London student and staff societies and bulletins. This broad recruitment strategy aimed to reach as diverse a group of participants, with differing levels of asthma severity, as possible. Participants were provided the option of completing the interviews at GP surgeries, hospital asthma clinics or Queen Mary, University of London.

### 2.2 Participants

Participants were purposively sampled to represent genders, different generational statuses (first, second and third) and ethnicity (Pakistani or Bangladeshi). Calculation of sample size in qualitative studies is determined as data collection takes place. We took an approach of recruiting until data saturation with respect to the research question, that is, when a comprehensive understanding of perspectives was achieved.^31^ Participants were included if they fulfilled the following inclusion criteria: (1) an active diagnosis of asthma, (2) aged 16 years or older and (3) of Bangladeshi or Pakistani ethnicity (self‐identification was checked by researchers). Participants' self‐reported generational status was reported in the expression of interest form and defined as follows^32^:

1. First generation (G1)—those born in South Asia or a country other than the United Kingdom and who settled in the United Kingdom.
2. Second generation (G2)—those born in the United Kingdom or arrived in the United Kingdom as a child (under the age of 10), and have at least one parent who was born in a country other than the United Kingdom.
3. Third generation (G3)—those born in the United Kingdom, where at least one parent was born in the United Kingdom or arrived in the United Kingdom as a child (under the age of 10), and grandparents who migrated to the United Kingdom having been born abroad.
4. Fourth generation (G4)—those born in the United Kingdom and at least one parent and one grandparent who were born in the United Kingdom.

### 2.3 Patient and Public Involvement (PPI)

PPI colleagues from Barts Health NHS Trust and the community were consulted to guide study design and to ensure that the study materials, in English and South Asian languages, were fit for purpose (including the written and oral participant information sheets detailing the scope of asthma self‐management, audio‐recorded and written consent forms, study posters, interview schedule and eligibility forms).

### 2.4 Data collection

An expression of interest form was completed by all individuals who showed an interest in the study. This asked individuals to indicate gender, age, ethnicity and generational status and complete three items (item 1, 3 and 20) selected from the Suinn‐Lew Asian Self‐Identity Acculturation scale (SL‐ASIA),^33^ validated for South Asian populations living in developed countries,^34^ to identify the degree of acculturation amongst participants. PPI feedback suggested that the use of the full validated scale was neither acceptable nor had face validity.^33^ Given that the scale was only utilized for purposive sampling, use of selected questions was considered acceptable.

The expression of interest form was used to purposively sample participants. Those eligible were asked to participate in an audio‐recorded semi‐structured interview. All participants were informed that data collected would remain confidential, anonymous and that they could withdraw from the interview at any time. Procedures recommended by Lloyd et al.^35^ were followed to achieve full informed consent if participants were not able to read, write or speak in English. An audio‐recorded patient information sheet and consent form on CD or online audio format was provided in the languages Sylheti, Urdu and English, along with written copies. Plans were in place for verbal consent to be audio‐recorded if participants could not provide initials or signatures. If needed, potential participants were given another chance to listen to the audio recordings before informed consent was taken in person by the bilingual researcher. Interview questions are presented in Table 1. Participants selected which language they preferred to speak in, and SA interviewed them (the primary researcher who is fluent in these languages; female; PhD student, health psychologist experienced in qualitative research).

**Table 1:** Interview schedule

1. Tell me about your asthma?
2. What does asthma self‐management mean to you? (If participants were unaware of asthma self‐management, a brief explanation was given)
3. What do you do to look after your asthma?
4. Tell me about your confidence in looking after your asthma?
5. What helps or influences the way you look after your asthma?
6. What do you think is the view of people in your community on asthma and how asthma is managed?

### 2.5 Data analysis

Interviews were digitally recorded. We transcribed interviews verbatim in the language chosen by the participants (Sylheti, Standard Bengali, Urdu and English); even if participants switched between different languages, and where necessary, data were back‐translated into English.^36^ Written communication strategies (‘Bengalish’, Bengali‐English and ‘Urlish’, Urdu‐English) that retain the pronunciation of South Asian languages but the words are written using the English Alphabet^36^, ^37^, ^38^ were used to transcribe South Asian languages because oral languages, such as Sylheti, have no written form, but are often translated in the closet dialect (Standard Bengali) and if then back‐translated into English, lose cultural, linguistic and contextual‐specific meanings and expressions that are important for analysis.^35^, ^39^

QDA Minor software was used to support data analysis. The data were analysed using thematic analysis guidelines suggested by Braun and Clarke.^40^ Table 2 lists the decisions underpinning analysis, as well as the steps taken for data analysis. To ensure consistency of interpretations, four researchers participated in independent coding of the data. SA coded all interviews, AD second coded the data (PhD student, an anthropologist experienced in qualitative research) and LS (an academic health psychologist) and HP (a clinical academic respiratory professor) group coded the data. The coding framework was compared, discussed and reviewed over time. Member checking was conducted to ensure the credibility of the interpreted data. Two participants contributed to this process, and the findings were reviewed by members of the PPI group to broaden perspectives.^41^ As there were no significant differences between the data obtained from the third‐ and fourth‐generation participants, and given the relatively small numbers in each group, these were grouped together.

**Table 2:** Decisions underpinning thematic analysis^40^

1.	Decisions were made on how to approach data analysis, which included using:
	(i) A contextualist method, which combines two methods: essentialist, which focusses on individual experiences, meanings and realities, and constructionist, which focusses on patterns of socially produced events, realities, meanings and experiences arising from structural conditions and/or sociocultural contexts. The combination of both methods considers the individual perspective on the meaning of experiences, motivations and interactions, and how this has an impact on the wider sociocultural influences on the production of these meanings and realities.
	(ii) An inductive ‘bottom‐up’ approach (without fitting data into a pre‐existing coding framework or analytical perception of the researcher).
	(iii) At a latent level, which describes the form and meaning of data at the surface stages (explicit level), and further detects, examines and interprets the underlying ideologies, assumptions and concepts, which are thought to shape these descriptions (interpretative level).
2.	Each transcript was read and re‐read for familiarity, where appropriate initial thoughts were noted.
3.	Initial codes were generated for all data manually. Initial coding was compared and discussed amongst researchers.
4.	Transcripts were entered into QDA minor for data management, and codes were electronically tagged to relevant data. All data were coded again. Codes from different researchers were compared and discussed.
5.	The long list of codes was re‐organized under potential themes. Mind maps were formulated experimenting with pieces of paper with written codes (with a brief description). An initial thematic map was generated based on the relationship between the codes and themes. Potential themes were discussed amongst researchers. Codes in QDA Minor were adjusted to reflect this initial thematic map.
6.	Themes were reviewed and refined, by checking them according to all the coded data extracts. If necessary, additional missed data were coded within the themes. The initial thematic map was adjusted to reflect these changes.
7.	Themes were further defined and refined, by writing a detailed narrative and analysis in relation to the research question, generating sub‐themes.
8.	The findings were written, along with illustrative examples of data.

### 2.6 Reflexivity

To maintain the quality of research, SA (the primary researcher) took reflexive notes in a research journal after each interview, which were discussed with the wider team, and peer briefings.^42^, ^43^ These strategies helped to raise researcher awareness of reactions and how ideas were imposed during the research process and upon the research.^43^ For instance, all participants were recruited from healthcare settings, with almost half interviewed in these settings. A few participants appeared to resent HCPs who they felt medicalized them. It is possible that these attitudes were displaced upon the researcher, who may have been perceived as an insider to the medical profession. The impact of the researcher standpoint was considered throughout the interpretation of the data.

## 3 RESULTS

### 3.1 Participant characteristics

A total of 27 participants consented to participate in the study (see Table 3). Interviews ranged from 20 min to over 2 h. Thirteen participants were Bangladeshi, and 14 participants were Pakistani. There were 14 females and 13 males, between the ages of 16 and 72 years, recruited from primary care (*n* = 15), secondary care (*n* = 11) and tertiary care (*n* = 1). One participant had severe asthma. There were 10 first‐generation participants, 10 second‐generation participants, 7 third‐generation participants and 1 Bangladeshi fourth‐generation participant. Most second‐ and third‐generation participants were diagnosed with asthma in childhood, including a first‐generation Pakistani participant (*n* = 12). The degree of acculturation in the sample revealed that (see Box 1; Table 3)^33^:

1. Twenty‐two participants identified themselves as British Bangladeshi and British Pakistani.
2. All participants could speak English to varying levels (basic, intermediary and fluent). Seven first‐generation participants spoke basic or little English. Spoken South Asian languages included Standard Bengali, Sylheti, Urdu, Punjabi, Hindu and Mirpuri.
3. Twelve participants identified themselves as bicultural. An additional nine participants identified themselves with a South Asian identity, of whom seven out of nine participants were from the first generation. Six further participants identified themselves as mostly westernized.

**Table 3:** Demographic characteristics

Generation	Ethnicity	Gender	Age	SL‐ASIA scale^33^		
				What language can you speak?	How do you identify yourself?	How would you rate yourself?
G1 First Generation *N* = 10	Bangladeshi	2 Female 3 Male	32–52	3 Mostly South Asian, Some English 1 Bilingual 1 Mostly English, some South Asian	2 South Asian: Bangladeshi 3 British Bangladeshi	2 Very South Asian 3 Mostly South Asian
	Pakistani	3 Female 2 Male	43–72	4 Mostly South Asian, Some English 1 Bilingual	2 South Asian: Pakistani 3 British Pakistani	2 Very South Asian 2 Bicultural 1 Mostly westernized
G2 Second Generation *N* = 10	Bangladeshi	4 Female 1 Male	21–49	2 Mostly English, some South Asian 3 Bilingual	5 British Bangladeshi	3 Bicultural 2 Mostly westernized
	Pakistani	2 Female 3 Male	41–49	1 Mostly English, some South Asian 4 Bilingual	5 British Pakistani	1 Mostly South Asian 4 Bicultural
G3 Third Generation *N* = 6	Bangladeshi	1 Female 1 Male	20–29	1 Mostly English, some South Asian 1 Bilingual	3 Mostly English, some South Asian 1 Bilingual	1 Mostly South Asian 1 Mostly westernized
	Pakistani	2 Female 2 Male	16–22	2 British Bangladeshi	1 British South Asian 3 British Pakistani	2 Bicultural 2 Mostly westernized
G4 Fourth Generation *N* = 1	Bangladeshi	1 Male	22	1 Mostly English, some South Asian	1 British Pakistani	1 Bicultural
	Pakistani	‐	‐	‐	‐	‐

### 3.2 Summary of themes

**Figure 1:**
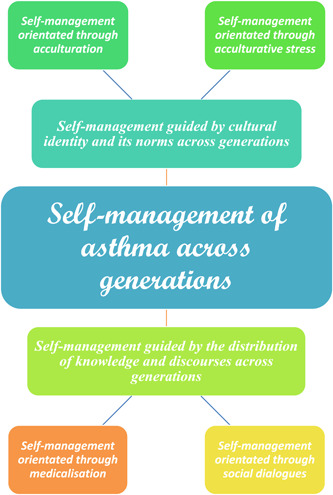
Thematic schema: Self‐management of asthma across generations

Data showed that there were intergenerational differences in asthma self‐management, which were contextualized, often because of factors such as complex cultural processes relevant for South Asians as members of an ethnic minority group, thereby leaving a variable impact on self‐management. Several sub‐themes emerged from the data that were collated into two main themes: (1) self‐management guided by cultural identity and its norms across generations and (2) self‐management guided by the distribution of knowledge and discourses across generations (see Figure 1).

### 3.3 Theme 1. Self‐management guided by cultural identity and its norms across generations

This theme illustrates that the awareness of relationships with others in different social spaces orientated how participants self‐managed asthma across generations. Second/third generations defined boundaries of cultural identity and its norms, which were negotiated, appraised and reflected upon. Enacted through acculturation and its stressors, this initiated a differential impact on self‐management. There were two subthemes.

#### 3.3.1 Self‐management orientated through acculturation

Some participants from the first generation believed that their home country was the better country for improving asthma because of the hot weather, unlike the United Kingdom, a ‘cold’ country. Some of these participants experienced better access to good‐quality paid healthcare, despite being developing South Asia countries with low resources.

I feel ‘erh, Bangladesh are more lucky. Bangladeshi people. If you pay, you see a good doctor. If you have money, you can have good treatment, but here I don't know what to do (laughs) (Bangladeshi, G1, male, aged 50)

Traditional beliefs about sweating influenced the self‐management of two first‐generation participants, who believed that they either needed to allow the body to sweat to rinse away impurities from inside the body or prevent the build‐up of sweat that could become cold with tissues or towels. In the former, continuous ablution helped him to remove impurities from sweating. Without these actions, it was believed that asthma could become worse.

Gham takhe ammar tanda aiyah asthma oi zai, ar shash khosto oi zai

From the sweat, my cold comes, and my asthma happens, and I get breathless (Bangladeshi, G1, female, aged 40)

The acculturation style of integration influenced how UK‐born generations (second/third) learnt, improvised and tested the boundaries of self‐management strategies, such as experimenting with Chinese CAM treatment on a trial‐and‐error basis. Continual use of these self‐management strategies was validated by beneficial experience. These strategies were considered strange and frowned upon by older first generations. Cultural endorsements transpired if this aligned with traditions or cultural norms relevant to the first generation. A male participant stated that there was little support for crossing cultural norms such as exercise, which was considered as novel, and this behaviour was questioned and criticized.

People are more ambitious I think, in the Bengali community I think people are ambitious only in a financial regard. If you're basically pursuing finance then everyone's very supportive, and they want in on it as well. Whereas, if you are saying you're going to have boxing match, you're crazy! If you tell someone you do like a military assault course, they'd be like, why would you do that? Why don't you just go around the park? Why you going to do a marathon for? Or flying planes for example, actually flying planes people love that, Bangali qoun, ‘Pilotor license anrai ni?’ [Bengalis say, ‘Are you get a pilot license?’] …You know typical Bengalis. But, sailing for example, why you going to sail for? Go Bangladesh and, I don't know. It's not as, because it's alien in the community a lot of things are alien and anything that's alien, is either frowned upon or ridiculed (Bangladeshi, 3G, male, aged 29)

Second/third‐generation participants also switched between personas in different contexts and adjusted self‐management accordingly. Individuals used traditional self‐management in familial settings (e.g., opting for wheezing instead of using the inhaler because of the stigma attached to being ill), compared to peer settings, where self‐management orientated from traditional to religious teachings (e.g., using the inhaler to look after oneself). A male participant described this contextual fluidity of self‐management below.

Comparing university to home. I mean to, if there's a family event, then I think people are more cultural and more back‐home minded. Even though we're here in the West, like when in those family groups, people take a step back into culture and they talk Bengali and this… But then the same people if they are in a university area, they'd be more open to it, and they'd be like okay like, it'd be looked at like more of a you know, under control thing (Bangladeshi, G4, male, aged 22)

The idea of living with asthma, including using the inhaler, was normalized, and accepted as the identity of UK‐born/raised generations (mainly the third generation).

My inhalers ‘erm, ‘erm, nothing it's just, literally my asthma, I've had it for so long so (laughs), I'm used to it now. It's just medicine (laughs) (Pakistani, G2, male, aged 41)

#### 3.3.2 Self‐management orientated through acculturative stress

Acculturative stress affected asthma self‐management throughout generations. To survive as an ethnic minority who migrated to the United Kingdom losing their social and economic capital, first‐generation participants prioritized dealing with relevant stressors over self‐management. Previous education/work credentials had little value, which meant settling for substandard housing and a lower employment status. For example, a first‐generation participant described how he had a better lifestyle in Bangladesh with a well‐paid job and benefits, but when he became a resident in the United Kingdom, he had to settle for a managerial retail job that required him to work in cold environments (around freezers), which worsened his asthma. His living standards were also poor in comparison to that in South Asia, such as damp housing with mould in the rooms, which exacerbated his asthma and caused asthma‐related mental health issues.

I am in depression, like you know I have, those things also because of my depression, stress, and this is the by‐product of European environment. We are, I am came here ah to change my conditions, but I could not change my conditions, rather, I make some by‐product. What I am earning here, I used to earn more than this while I was in Bangladesh. I am paying you know, in my Bangladesh, in Dhaka, if I discuss with my friends, that you see, my house size is, I think is a bit bigger than your store size, but I am living in a small room, but how in Bangladesh our you know, toilet is like this (laughs) (Bangladeshi, G1, male, aged 50)

Another participant emphasized that the UK environment has given him considerable stress that led to psychological issues such as depression, distracting him from concentrating on his asthma. He blamed the government for stopping him from being with his family from Pakistan, who provided support for his asthma, by not giving permission to bring them to the United Kingdom. There were also examples of resistance to asthma advice given to them from children and grandchildren raised in the United Kingdom, since it did not comply with their way of understanding asthma self‐management.

Second generations, on the other hand, had to contend with traditional expectations of others over practicing self‐management, playing on female gender roles and norms, such as taking care of domestic responsibilities (including those for their extended families), expecting women to be good mothers, not to smoke (as men smoke) and not to socialize at night because they need more protection than men. Often, this produced acculturative stress and cultural conflict that worsened asthma. Women prioritized religious beliefs about self‐management over traditional culture, such as treating the body well and trusting God to help them cope with asthma, to justify acting against traditional gender roles that compromised self‐management. Other second generations, as illustrated in the quote below, had to battle with stressors such as the first generation enforcing their ideas about healthy food as good asthma control.

So, there's always that battle between, our generation and our, parent's generation. ‘Erm there's the battle in terms of, listening to people, there's the battle in terms of what you eat as well. We can't live the lifestyle that they have lived. They've lived a more, labour, hard work lifestyle, very little for reward, yeah. We get a lot more reward, from work, or we can't eat, and just sit down and just, nap, nap, nap all day like they do (laughs). ‘Erm… So, that's why we have a slightly different life to them as well. We, they wouldn't like to go out and eat, with, we would go out and eat. Yeah, not all the time, but it's nice like once in a while to go out and eat, they wouldn't like it at all. So, there's always kind of, ego within our own communities, and in our own families there's always that kind of culture clash, a generation clash of how we want to do things, compared to how they want to do things [referring to self‐management strategies] (Pakistani, G2, male, aged 42)

In comparison, females of the first generation believed that it was an obligation to fulfil their roles (as a mother and daughter) and perform domestic chores (cook fresh curries every day, wash the dishes) and stay at home (attributed to being a good mother), even though this worsened asthma symptoms such as feeling breathless or tired. Compromises were made to complete domestic chores such as covering the face with a scarf and using candles to avoid cooking fumes, despite being unable to enjoy the fruits of these tasks; for example, they could not eat some food they prepared as it could worsen their asthma. They delegated those physical domestic roles that they believed men could take over such as hoovering, grocery shopping and taking children out to social activities. Some mothers refused to delegate domestic tasks to children due to the belief that children need to focus on education. The absence of duty because of grown up children and less traditional demands was described by one participant.

Just relax it, yes. Then I feel okay. I have, no any duty (laughs), only husband (laughs). He helped me. His ‘erh, his a, his good, because a lot of men, they are always really terrible, ‘I want coffee, I want tea’ (laughs). ‘This is my, dinner time, or look at time’ (laughs). It's very hard duty (Pakistani, G1, female, aged 72)

The importance of acculturative stress in impacting self‐management was highlighted through comparison to White populations, who were believed to be better at self‐management. Without the culture‐specific stressors that UK‐born South Asians face, they have more capacity to be active in learning about and treating their asthma, they are perceived to receive more social support and can prioritize self‐management. Collective cultural traditions among participants in this study for dealing with problems and family‐specific etiquettes and responsibilities (e.g., looking after and respecting elders) reduced the possibility of focussing on asthma self‐management and compromises made that worsen asthma.

We tend, unfortunately to have a lot of stressors [referring to stresses that trigger asthma] in our Asian, families, whether it's the house that you're living in, whether it's the kids or parents or extended families. I mean, one issue, one issue would go around that home; family, and everybody gets involved and everybody gets stressed about something, and everyone has to be like, kind of, ‘erm so, we tend to find out within our own communities. There is a lot more stress than, ‘erh non‐Asians. To the point where you sometimes, you even say that a ‘Ghoreh’ [a white person] they don't deal with any of this rubbish. Yeah definitely, I think because they have a lot less things that they worry about and stress about (Pakistani, G2, male, aged 42)

The third/fourth generations sometimes had CAM imposed on them by their parents/grandparents as a child to treat their asthma. This created intergenerational conflict.

So, the spiritual healing, what it is was, you just have like water and, mint tea or something and, you listen to few stuff and it was something like that. I done it few, I think once or twice I done it. I got forced into it… It's something yeah, it is a bit religious, I think. It was like, the first time I done it in this house, the, some guy who came, and he had like little sweets and stuff and he read like Qur'anic ayahs [verses] and stuff, my grandma like said, ‘You need to get fouw done [blown on] or something’ (laughs). So, I was like, I didn't know any better at the time, and then he done something. I don't know if it helped or not, and then he gave me a few stuffs to eat and this and that, and then I didn't taste it, or I didn't finish it. And then she [grandma] goes; ‘Oh, because I didn't finish it, now it didn't work or something, whatever’… (Bangladeshi, G4, male, aged 22)

### 3.4 Theme 2. Self‐management guided by the distribution of knowledge and discourses across generations

This theme illustrates available information/discourses on asthma and self‐management from the community and healthcare services. Enacted through social dialogues and medicalization, this orientated how participants made sense of asthma self‐management across generations. There were two subthemes:

#### 3.4.1 Self‐management orientated through social dialogues

The extent to which participants engaged in conversations about asthma with others shaped self‐management, including illness beliefs and the provision of social support. Participants from the first and second generation believed that asthma and its discussions were less important and common in the community than those concerning other illnesses such as diabetes and epilepsy.

If you're starting to feel asthmatic and you take your pump and then you calm down, it's kind of the end of it, so it finishes there, like there's no problem but with something else, if you're, say if you have epilepsy or something like that, if you have a fit, then there's a lot more to it than just take a pump and then it's kind of finished, does that make sense? That's why I don't feel like it is talked about, because it's so just normal… (Pakistani, 3G, male, aged 29)

As a result, most family support centred on practical assistance when asthma symptoms were noticed such as calling the emergency services.

Only one of my aunts, who, she… Well actually I don't speak to her about it unless it's, for example, a few times when the inhalers weren't working, so she would call the ambulance and that's when they would say, ‘Oh, is it purple? This and that… It's like you know, when someone calls an ambulance, it's like something serious… (Bangladeshi, 4G, male, aged 22)

While asthma remained a rare topic to discuss socially, some second/third generations believed that they could discuss asthma in terms of certain subjects, such as comparing inhalers, if another close person had asthma, and if the person was considered knowledgeable.

No I think I know a few of my friends, but them, they don't need the medicine every day like me… It would just be about, the medication, what pumps they have and which ones I have, that would be about it, but we don't talk about how we deal with it or anything (Bangladeshi, 3G, aged 20)

#### 3.4.2 Self‐management orientated through medicalization

Most individuals, particularly first generations, heavily relied on self‐managing their asthma. They drew on medical information (in the absence of knowledge about asthma) provided and reinforced by HCPs, placing emphasis on how and when to take medication rather than why medication was necessary. Therefore, self‐management from this perspective was simplified to medication adherence and symptom monitoring, leading individuals to perceive that no additional self‐management support was needed.

Just don't see it [asthma self‐management] as an issue really. Like if you got a headache you take paracetamol, you know. (Bangladeshi, G2, female, aged 34)

Intergenerational differences in the medicalized self are detailed in Table 4. The first generations, who were typically older, were ‘ideal’ medical patients because they strictly adhered to medication and rarely sought medical attention, so they preferred to wait it out at home. One reason surmised for this was that they are familiar with habits of people from Bangladesh and Pakistan when it comes to approaching healthcare facilities.

50 years ago, there was, probably no treatment. Definitely, no treatment in rural Pakistan. So, people will have to live with it. In Pakistan, it's, I don't think so. They need more years. It's the way they are taught or the way they are brought up. They can't see, in that much health education in Pakistan or social care in Pakistan. So, I don't think there will be any significant change in people, managing chronic illness. Other, there is more, dependence on family then here, because normally state intervenes in England. The state does not intervene in Pakistan. So, people have to look after their elders. (Pakistani, G1, male, aged 56)

**Table 4:** Generational differences in perceived medicalization of the self that influence asthma self‐management

Generation	Levels of medicalization
Older first generations	Strict medication adherence, on time Followed advice from HCPs Medication believed to be a solution for all illnesses Prefer to be alone during worsening of asthma, e.g., rest/sleep; reluctance to speak about asthma and seek familial/medical aid Adherence to CAM alongside inhalers
Other first generations	Adherent to medication Sought medical aid
UK‐born generations	Adherent to medication but cautious about medication decisions Sought medical aid Discussed asthma when it was necessary
Across generations	*De‐medicalization*: Medical instructions and treatment were questioned Active seeking of other sources of information Preference for HCPs to provide individualized supported self‐management Some distrust for following all HCP opinion/advice Belief that self‐management should be holistic based on other preventative factors rather than solely based on medicine

The younger first generations adhered to medication but sought medical advice earlier. UK‐born generations adhered to medication, but took precautions against side‐effects (actively choose which medication to take) and discussed asthma if it was necessary. When information was sought from other sources, the medicalized self was contested by a few individuals (the de‐medicalized self; see Table 4). Instead of a generic medical approach to asthma, these individuals believed that HCPs should recognize that asthma is heterogeneous, and that holistic tailored care is required.

I don't really need this… The spacer, and ‘erh, the generalisation of the part of asthma professionals is slightly… They shouldn't be making too much effort on generalisation as, ‘You should do this’. Same thing does not fit everyone. I use it [the spacer] for my son because he can't inhale properly. After 30 years of yoga, I have very precise control of my breathing. So, but the geniuses, nobody would agree, or nobody want to admit that. Don't generalise things. Don't force. (Pakistani, G1, male, aged 56)

## 4 DISCUSSION

### 4.1 Main findings

In this study, asthma self‐management varied according to generation. This was itself influenced by complex sociocultural and contextual processes, such as acculturation, which are particularly relevant for UK South Asians as part of an ethnic minority group. This warns against making broad generalizations simply based on Bangladeshi and Pakistani cultural backgrounds. Self‐management strategies specific to the first generation included choosing to travel to South Asia to treat asthma (through the hot weather and paid care in health services) and sweating out impurities from the body. The UK‐born/raised generations learnt and experimented with self‐management practices that were not always in line with their traditional culture but included those from other cultures. This more integrated approach was often frowned upon by older first generations. For later generations, self‐management was also fluid according to whether the person was in family settings, adhering to traditional attitudes to minimize the severity of asthma, or peer settings, using westernized ideals or religious teachings to look after one's asthma. Asthma had a relationship with acculturative stress, which influenced the priority given to self‐management throughout generations. First generations struggled with their asthma because of stressors related to migration, such as the loss of socioeconomic capital and adaptation to the new environment, where depression and stress were described as common byproducts of this struggle. Cultural conflict was a challenge for second generations, who struggled with traditional expectations particularly around gender roles, such as women's responsibility for domestic chores over self‐managing asthma, and norms of categorizing ‘healthy’ foods such as roti to treat asthma, all of which risk worsening asthma. Whilst third/fourth generations had more independent attitudes to self‐management, during childhood, more traditional strategies can still be imposed as illustrated by CAM use by their parents/grandparents to treat their asthma. Frequently, this created an internal conflict. Self‐management was also based on the amount and type of available discourses on asthma, within the community and HCPs. Discussions with HCPs, for instance, created a platform to medicalize self‐management by framing it as medication adherence and symptom monitoring. The extent of medicalization differed across generations.

### 4.2 Interpretation of findings

The simplistic approach of tailoring cultural interventions solely through adaptations such as language modifications of resources^12^, ^13^, ^14^ is challenged by the findings of this study, which reinforce that culture and self‐management should not be treated as static.^6^, ^7^, ^8^ South Asians are not a homogeneous cultural group, but nurture distinct intergenerational strategies for asthma self‐management, with adaptation through processes such as acculturation, illustrating that self‐management needs to be socio‐culturally and contextually relevant.^8^, ^9^, ^44^ Integration promotes bi‐cultural competences and identities, including a good grasp of English, meaning that we found that participants tended to learn, negotiate and experiment with and/or fuse self‐management strategies from different sources (traditional culture, other cultures and religion).^18^, ^19^, ^45^ What constitutes good self‐management is continually defined, agreeing with previous studies with different communities that gave precedence to religious self‐management over traditional cultural strategies, including African Americans,^46^ American Indians^47^ and South Asians.^48^ The first generation used distinctive strategies in line with their traditional culture, as illustrated by the belief that the hot weather of South Asian countries can help/treat asthma. This can be explained by traditional South Asian treatment beliefs around heat. Hot and cold beliefs suggest that cold causes, triggers and sustains illnesses, and therefore needs to be balanced by using heat or hot remedies and avoidance of anything considered to be cold.^49^, ^50^, ^51^ Sweating to improve asthma also aligns with cleansing belief models, which stipulate that the removal of impurities from the body improves health.^49^, ^52^

The priority given to coping with acculturative stress rather than asthma self‐management resonates with previous literature showing that intergenerational conflict occurs with a wide range of health behaviours such as tobacco use,^53^ atherosclerosis,^54^, ^55^, ^56^ breastfeeding practices,^57^ cardiovascular illness,^58^ musculoskeletal pain^59^ and body image and eating attitudes.^34^ For instance, a study by Choudhry and Wallace^57^ showed that generational clashes were found between younger‐generation South Asian mothers and their first‐generation mothers‐in‐law, who preferred formula feeding compared to breastfeeding. Effective tailored intervention might need to be multilevel, incorporating both the family and the individual. We also found generational clashes in gender expectations. There has, however, been little research in this area other than looking at the asthma trigger exposures related to traditional gender‐related occupations and roles.^60^, ^61^ For instance, exposure to gas cooking, chemicals in cleaning, hairdressing and salon‐related labour have been demonstrated to trigger asthma symptoms such as airway inflammation, allergies and lung function. Traditional male occupations, such as carpentry, auto‐spray painting and baking, expose one to wood dust, cooling lubricants and flour.^60^, ^62^, ^63^, ^64^ Acculturative stress seems to be higher in the first generation, who were less acculturated to their new environment, describing psychological distress such as ‘depression’, compared to the third generation.^59^ This may be because previous studies have found that integration was highly correlated with better health outcomes for various cultures, including Hispanics and Japanese.^24^, ^25^, ^65^

Family support was fragmented and limited to when asthma symptoms were observable, agreeing with previous research that found that support provisions were primarily restricted to incidences of asthma attacks or when there was a need for language translation.^66^, ^67^, ^68^, ^69^ However, the level of support from the immediate family for other illnesses (e.g., diabetes) was much higher.^70^ It is unclear why there was more social awareness, acceptability, discourse or social support for other illnesses compared to asthma, but it can be related to perceptions of the seriousness of asthma.^71^ The finding that there was more knowledge about medication than about asthma as a condition is concerning, especially in UK‐born generations, who are fluent English speakers, and challenges previous studies,^48^, ^68^ including systematic reviews^12^, ^72^, ^73^ that found that information on medication was difficult to digest for South Asians. We found problems for retaining information related to using peak flow monitoring devices, which should be addressed in future interventions. Holistic exploration of self‐management, focussing on biopsychosocial factors, seems to be largely overlooked by HCPs, agreeing with previous research that suggests that medical professionals feel unequipped to deal with psychosocial matters and the need to prioritize this within time‐limited consultations.^44^, ^74^ A systematic review synthesis by Joseph et al.^75^ found that the provision of knowledge by itself from HCPs did not mean that people had the power to make decisions. Patients can become passive in doctor–patient encounters and are less likely to question them; for example, they might allow HCPs to make decisions for them because they want to be perceived as the easy/good patient. Resistance against medicalization occurred due to receiving other information about asthma, as in this study, along with limited community discussions, suggesting that if individuals were fully informed about asthma and self‐management, it can transform attitudes towards medicalization. Higher levels of medicalization in older first generation individuals can be linked to habits developed around routines and past experiences of healthcare in native countries, where shared responsibilities of self‐management are not fully promoted.^76^

### 4.3 Implications for policy, practice and research

The cultural context of a person's life affects their perspective and self‐management of their asthma. This study warns against simple cultural stereotyping. Current strategies that incorporate culture into self‐management interventions commonly involve ‘modified’ top‐down approaches, taking an existing intervention from one setting to deliver it to another population, through one‐off translations of materials and use of culturally relevant images, thereby homogenizing cultural groups. This is likely to be an inadequate and unprogressive strategy, resulting in interventions with limited effectiveness.^12^, ^14^ In this study, our findings emphasize that cultural diversity further evolves across generations, shaping asthma self‐management behaviours and producing an intricate challenge for researchers. ‘Targeted’ or ‘tailored’ bottom‐up interventions can help met these challenges. The former accounts for shared group characteristics of a cultural group, such as cultural beliefs and the process of acculturation, while the latter considers cultural dimensions unique to individuals within a group, such as the level of religiosity.^12^, ^14^, ^77^, ^78^ Tailored exemplar interventions are rare in asthma self‐management, illustrating that progress has been limited. There is some evidence of effectiveness for targeted interventions that have considered cultural relevance, by co‐developing resources with members of the community and/or HCPs and pilot testing in focus groups for clarity, relevance and acceptability, and refined before evaluation.^12^ Crucially, incorporating these components into the development process can allow information to become more relevant and acceptable in a population.^13^, ^79^ For instance, Poureslami et al.^80^ incorporated cultural gestures, humour, storytelling and social interaction styles by a community member and/or clinician in educational videos on asthma self‐management appropriate for Chinese and Punjabi Indians, allowing participants to develop trust and credibility of the delivered intervention.

Guidelines or frameworks for developing interventions considering culture are limited and are an area that needs further attention, as are guidelines that promote cultural competence in HCPs and healthcare services.^6^, ^7^, ^8^ To promote biopsychosocial skills and self‐management support in cross‐cultural situations, and avoid unintended consequences of medicalization, the incorporation of cultural and practical strategies could be useful, including employing staff to reflect the diversity of the community, the use of bilingual interpreters and cultural and linguistic trajectories.^44^, ^74^ There is a wider need for self‐management education for participants that should be available in healthcare, community and remote digital technology services.

### 4.4 Strengths and limitations

Few studies have sought to understand asthma self‐management in South Asians with a generational lens.^81^ Recruited participants were from different parts of the healthcare service with varied experiences and perceptions of asthma self‐management, although, in most first generation participants, recruitment was predominantly from secondary care, which can reflect the patients' consulting behaviour and/or the severity of their asthma. The breadth of our recruitment strategy enhances transferability to other similar settings for South Asian generations and other ethnic minorities. Despite our efforts, the adopted community recruitment approach was not effective. We must therefore be cautious in generalizing our findings to individuals from the third/fourth generation or who do not access healthcare systems for their asthma. It was a strength of the study that participants were able to participate regardless of the language spoken. Some participants chose to express themselves in their preferred language in interviews, even though they may speak English, and other generations switched between languages mid conversation, allowing cultural expressions to create certain meanings that cannot be articulated in English.^82^ Data transcription and analysis in the original language spoken by participants (alongside English) helped avoid common criticisms of back translations,^83^ and addressed cultural and linguistic gaps in transcribing data,^38^, ^84^ and was a strength of the study. Having a researcher from the population of interest was a further strength.

## 5 CONCLUSION

Cultural diversity has a strong influence in shaping asthma self‐management, especially across generations and context, showcasing an important area for developing congruent guidance in tailoring interventions and implementing cultural competence strategies in healthcare services. Future research should build on this study and explore how HCPs provide self‐management support to these communities while accounting for their cultural differences.

## AUTHOR CONTRIBUTIONS

*Development of concept and design of the work and critical revision of the article*: Salina Ahmed, Liz Steed and Hilary Pinnock. *Data collection and initial draft of the article*: Salina Ahmed. *Data analysis and interpretation and final approval of the article version to be published*: Salina Ahmed, Anna Dowrick, Liz Steed and Hilary Pinnock.

## CONFLICT OF INTEREST

The authors declare no conflict of interest.

## Data Availability

The data that support the findings of this study are available on request from the corresponding author. The data are not publicly available due to privacy or ethical restrictions.
